# Knowledge, attitudes, and practices in adult patients and parents of pediatric atopic dermatitis patients: a cross-sectional study

**DOI:** 10.3389/fpubh.2024.1460044

**Published:** 2024-11-14

**Authors:** Zhifeng Nie, Pengyang Fan, Yuting Zhou, Sheng Han

**Affiliations:** ^1^School of Pharmaceutical Sciences, Peking University, Beijing, China; ^2^International Research Center for Medicinal Administration, Peking University, Beijing, China; ^3^Medical Policy Research Center, Beijing Health Promotion Association, Beijing, China; ^4^School of Nursing, Wuhan University, Wuhan, China

**Keywords:** knowledge attitude and practice, atopic dermatitis, influencing factor, questionnaire survey, dermatovenereology

## Abstract

**Objective:**

To investigate the knowledge, attitude, and practice (KAP) of adult Atopic Dermatitis patients and parents of pediatric atopic dermatitis patients toward the disease.

**Methods:**

A cross-sectional study was conducted from January to February 2022, involving 1,193 Asian Atopic Dermatitis patients from the Atopic Dermatitis patient organization “Atopic Dermatitis Home.” The study included 594 adult patients and 599 parents of pediatric atopic dermatitis patients, with self-designed questionnaires assessing general demographics and continuous variables KAP.

**Results:**

Adult patients demonstrated higher knowledge scores, particularly in areas of bathing and skincare (3.64 ± 0.68 vs. 3.35 ± 0.93, *p* < 0.01), and treatment precautions (3.35 ± 0.93 vs. 3.81 ± 0.51, p < 0.01), compared to parents of pediatric patients. In terms of practices, adult patients scored higher in treatment adherence (0.69 ± 0.96 vs. 3.33 ± 1.19, *p* < 0.01) and lifestyle management (1.85 ± 0.39 vs. 1.69 ± 0.59, *p* < 0.01), while parents scored higher in risk avoidance (1.58 ± 0.79 vs. 1.62 ± 0.88, *p* < 0.01). Regression analysis revealed that knowledge and attitudes significantly positively affected practices. Treatment adherence was positively associated with knowledge of treatment precautions (B = 0.323, 95% CI 0.175, 0.471, *p* < 0.001) and negatively with disease characteristics (B = -0.112, 95% CI -0.216, −0.008, *p* = 0.035). Self-treatment was positively associated with knowledge of disease characteristics (B = 0.154, 95% CI 0.036, 0.272, *p* = 0.011) and medication attitudes (B = 0.282, 95% CI 0.208, 0.356, *p* < 0.001). Lifestyle management was positively associated with knowledge of skincare (B = 0.071, 95% CI 0.036, 0.106, *p* < 0.001) and treatment precautions (B = 0.160, 95% CI 0.096, 0.224, *p* < 0.001), but negatively with patient type (B = -0.127, 95% CI -0.184, −0.070, *p* < 0.001). Risk avoidance was positively associated with knowledge of skincare (B = 0.128, 95% CI 0.067, 0.189, *p* < 0.001) and treatment precautions (B = 0.163, 95% CI 0.053, 0.273, *p* = 0.004).

**Conclusion:**

The study concluded that knowledge and attitudes significantly affect health practices among Atopic Dermatitis patients. Enhancing patient education on treatment precautions and skincare can improve adherence and management behaviors, highlighting the need for targeted educational interventions.

## 1 Introduction

Atopic dermatitis (AD), also known as atopic eczema, is a common chronic skin disorder. It is characterized by recurrent eczematous lesions and pruritus. AD can affect individuals at any age, with over half of pediatric patients presenting symptoms within the first year of life, and often persisting into adulthood (1, 2). However, adult-onset AD is also prevalent, with approximately one-quarter of adult AD patients experiencing initial symptoms in adulthood (3). In China, the prevalence of AD is estimated to range from 14.94 to 30.48% among children and approximately 4.6% among adults (4–6). The chronic and relapsing nature of eczema, severe itching, clinical manifestations, comorbidities, and skin complications associated with AD significantly diminish the quality of life for patients and their families. These symptoms lead to sleep disturbances and decreased work or academic productivity and negatively impact emotional and social well-being (7). Long-term AD is associated with depression, anxiety, and suicidal tendencies (8).

As a chronic and recurrent disease, AD patients require long-term treatment. Non-pharmacological interventions, including moisturizers, bathing practices, and wet wrap therapy, in conjunction with pharmacological management aspects such as dosage and frequency of application, collectively impact the treatment efficacy and control of the disease (9). Patients with a comprehensive understanding of the disease and its treatments generally exhibit reduced disease severity (10). However, the current state of AD management remains inadequate. Parents of pediatric AD patients and adult AD patients often lack knowledge regarding the pathogenic factors and specific medical terminology, such as the ‘fingertip unit,’ related to the disease and its treatments, leading to inadequate disease management. Attitudes towards treatment also play a crucial role in disease management; for instance, corticosteroid phobia often leads to poor treatment adherence (11–13). These factors collectively contribute to the challenges in managing AD, making it difficult for many patients to achieve effective disease control.

Given the severity of the disease and the widespread inadequate management, developing strategies to enhance patient knowledge and coping mechanisms are increasingly important. Research by Sokolova et al. (14) indicated that lack of knowledge, patient dissatisfaction, and frequency of follow-up are factors contributing to poor AD treatment outcomes. Therefore, it is necessary to understand AD patients’ knowledge, attitudes, and practices regarding the disease. The Knowledge-attitude-practice (KAP) theory, which combines cognitive and social psychological principles to explain the relationships between the three levels of health behavior change (15), is widely used in the medical field to study and intervene in various health-related behaviors (16–19). Utilizing this theory for investigation can provide a comprehensive reflection of AD patients’ cognition (20). In other disease domains, KAP has also been used to study how knowledge and attitudes influence practices (21–23). In recent years, KAP research in the context of AD has predominantly been conducted by many countries, with most studies addressing only one or two aspects of knowledge, attitudes, or practices. Reljić et al. (20) conducted the only complete KAP survey in the domain of AD in Serbia during 2015–2016, involving parents of pediatric AD patients. The survey revealed that older parents, married or partnered, and those with personal experience of AD demonstrated a higher level of knowledge about the condition, while older and employed parents had stronger positive attitudes toward their children with AD. Parental supportiveness in managing children with AD was positively associated with older age and inversely associated with higher educational attainment. However, this study included only families of pediatric patients and did not explore the entire AD patient population, nor did it investigate the relationships between the three factors. Currently, there is a lack of related research in China, posing challenges in assessing the current state of disease cognition among Chinese AD patients and implementing patient-specific educational interventions to improve treatment outcomes. This study aims to utilize a cross-sectional research method to investigate knowledge of the adult AD patients and the parents of pediatric AD patients and examine the association between knowledge, attitudes, and practices.

## 2 Materials and methods

### 2.1 Samples

This cross-sectional study was conducted from January to February 2022. The survey was administered online to AD patients through the AD patient organization “Atopic Dermatitis Home” (hereinafter referred to as “AD Home”). This study includes Asian patients diagnosed with AD for at least 1 year, defining pediatric patients as those under 18 and adults as those over 18. Questionnaires for pediatric patients (under 18) were completed by their parents. Based on the formula $n=\frac{Z_{\frac{\alpha }{2}}^{2}p\left(1-p\right)}{E^{2}}$ , with an AD prevalence of 14.94% in children and 4.6% in adults (4–6), using a 95% confidence level and a 3% margin of error, the calculated sample sizes for children should be above 543 and 188 for adults. A total of 1,207 questionnaires were distributed, with 7 excluded having no responses. A total of 1,200 patients were surveyed, including 600 adult patients and 600 pediatric patients. Participants who lived outside China at the time of completing the survey and those with data logic errors were excluded. Hence, the final sample included for analysis was 594 adult patients and 599 pediatric patients. All participants provided informed consent electronically before participating in the study. The study was approved by the Biomedical Ethics Committee of Peking University, with ethics review approval number IRB00001052-21137.

### 2.2 Data collection

The questionnaire for this study comprised four independent sections: (i) General demographic information: including age, gender, and household annual income in the past year. (ii) Knowledge; (iii) Attitude; and (iv) Practices related to AD.

Since there are no existing questionnaires specifically designed to assess the knowledge, attitudes, and practices of adult AD patients and the parents of pediatric AD patients, a new questionnaire was developed based on the study’s objectives. This study included multiple evaluation items for each dimension—knowledge, attitudes, and practices—serving as observed variables. The sum of the scores for each of the three dimensions reflects a quantitative assessment of knowledge, attitudes, and practices. A basic questionnaire was developed after summarizing 35 articles that included at least one of the dimensions (knowledge, attitudes, practices). This basic questionnaire was then evaluated by 19 clinical dermatologists. Following two rounds of semi-structured interviews with dermatology experts, the final questionnaire was established (Table 1). The Cronbach’s alpha for the knowledge, attitudes, and practices sections were 0.656, 0.652, and 0.685, respectively, indicating acceptable internal consistency. Structural validity was tested using factor analysis, with KMO (Kaiser-Meyer-Olkin) values of 0.797, 0.598, and 0.783 for the three sections, all above 0.50, allowing for factor analysis. The significance of Bartlett’s test of sphericity was *p* < 0.01, indicating significant correlations among items, thus confirming the suitability for factor analysis. The factor analysis resulted in a final questionnaire comprising 27 items.

**Table 1:** Final survey questionnaire.

Dimension	Code	item wording	Options
Cognition	K1	Climate change, lifestyle changes, improper bathing, infections, and allergen exposure can trigger atopic dermatitis	Do not know, Disagree, Agree
	K2	Psychological factors (e.g., stress, anxiety, depression) play a role in the onset of atopic dermatitis	Do not know, Disagree, Agree
	K3	Atopic dermatitis is not a contagious disease	Do not know, Disagree, Agree
	K4	Atopic dermatitis is usually hereditary	Do not know, Disagree, Agree
	K5	Skin itching is usually caused by dryness	Do not know, Disagree, Agree
	K6	Neutral to low pH, hypoallergenic, fragrance-free soap-free cleansers should be used for bathing	Do not know, Disagree, Agree
	K7	Bath water temperature should be kept lukewarm, and bathing time should be limited to 5–10 min	Do not know, Disagree, Agree
	K8	Emollients (ointments or creams) should be applied generously and frequently, immediately after bathing while the skin is still damp	Do not know, Disagree, Agree
	K9	After bathing, the skin should be gently patted dry or allowed to air dry rather than being rubbed dry	Do not know, Disagree, Agree
	K10	Showering/bathing frequency should be daily or every other day	Do not know, Disagree, Agree
	K11	If prone to skin infections, diluted bleach baths can be used	Do not know, Disagree, Agree
	K12	The treatment of atopic dermatitis is a long-term, chronic process	Do not know, Disagree, Agree
	K13	Following medical advice can minimize the occurrence of adverse drug reactions	Do not know, Disagree, Agree
	K14	Topical corticosteroids are the first-line medication for atopic dermatitis	Do not know, Disagree, Agree
	K15	Mechanical and chemical irritants to the skin should be avoided in daily life	Do not know, Disagree, Agree
	K16	Before eliminating foods, it is necessary to establish a causal relationship between the food and the outbreak	Do not know, Disagree, Agree
Attitudes	A1	I believe the treatment of atopic dermatitis is a long-term, chronic process	Yes, No
	A2	I believe treatment should be strictly according to the doctor’s advice	Yes, No
	A3	I am willing to get the doctor’s help to maintain treatment during remission periods	Yes, No
	A4	I believe that daily skin care does not need to be strictly adhered to when not having a flare-up	Yes, No
	A5	I believe that keeping indoor air clean and dry is not very necessary	Yes, No
	A6	I believe it is unnecessary to significantly change my diet to avoid food allergies	Yes, No
	A7	I believe avoiding all potentially allergenic foods is beneficial for disease treatment	Yes, No
	A8	The side effects described in medication instructions make me worried about using the medication	Yes, No
	A9	I worry that using medication or other treatments will have negative effects on my body	Yes, No
	A10	I am afraid of using hormones	Yes, No
Behaviors	P1	Strictly follow the prescribed dosage by the doctor	Yes, No
	P2	Strictly follow the prescribed intervals by the doctor	Yes, No
	P3	Follow the prescribed number of doses by the doctor	Yes, No
	P4	Use the medication prescribed by the doctor or the treatment method suggested by the doctor	Yes, No
	P5	Purchase and use medication based on friends’ recommendations, advertisements, or personal experience	Yes, No
	P6	Strictly follow the doctor’s requirement for regular follow-ups	Yes, No
	P7	Reduce or stop medication when I feel my disease is under control	Yes, No
	P8	Reduce or stop medication when experiencing side effects	Yes, No
	P9	Increase medication when symptoms worsen	Yes, No
	P10	I pay close attention to self-management even when my condition is stable or asymptomatic	Yes, No
	P11	I take immediate measures when I cannot avoid contact with or consumption of allergens	Yes, No
	P12	Use synthetic fiber pillows and non-breathable mattress covers	Yes, No
	P13	Avoid fabric or leather furniture, plush toys, carpets, and pets at home	Yes, No
	P14	Maintain indoor air filtration, circulation, and dryness	Yes, No

In the final questionnaire, knowledge-related questions were divided into three dimensions: disease characteristics, bathing and skincare, and treatment precautions, totaling 11 questions. Respondents chose from three options: agree, do not know, and disagree. Agree responses were scored one point, while do not know and disagree responses were scored zero points. The knowledge score ranged from a minimum of 0 to a maximum of 11.

Attitude scores ranged from 0 to 3, assessing respondents’ attitudes toward medication, specifically regarding side effects, negative impacts on the body, and attitudes toward steroids. Respondents answered yes or no; negative responses were scored one point, while positive responses were scored zero points. The attitude score ranged from a minimum of 0 to a maximum of 3.

Practice-related questions covered four dimensions: treatment adherence practices, self-treatment practices, lifestyle management practices, and risk avoidance practices, totaling 13 questions. Respondents answered yes or no; positive responses were scored one point, while negative responses were scored zero points, except for questions p5, p7, p8, and p9, which were reverse scored. The practice score ranged from a minimum of 0 to a maximum of 13.

### 2.3 Analysis of data

Based on the research objectives and the completeness and validity of the collected data, IBM SPSS Statistics for Windows, Version 27.0 (IBM Corp., Armonk, NY) was used for statistical analyses. Descriptive statistics for continuous variables were presented as mean and standard deviation, while categorical variables were described using frequency and percentage. Independent sample t-tests and non-parametric tests were used to compare the association between different demographic characteristics and knowledge, attitudes, and practices. A significance level of *p* < 0.05 was considered statistically significant. The impact of patients’ knowledge and attitudes, along with general demographic information, on practices was analyzed using linear regression. Collinearity diagnostics showed that all variables in the linear regression had VIF values less than 10, indicating no multicollinearity among the independent variables.

## 3 Results

### 3.1 Sample description

A total of 1,193 patients were included in this study, comprising 594 adult patients and 599 pediatric patients. The data for pediatric patients were provided by one of their parents. Table 2 shows the classification of participants involved in the study based on their demographic and health data. The average age of the adult patients was 31.97 years, while the average age of the pediatric patients was 5.50 years. The proportion of female patients was 28.59% among adults and 47.58% among pediatric patients. The highest educational attainment for adult patients was predominantly undergraduate/college level, accounting for 71.04%, whereas the majority of pediatric patients were in elementary school or below. The majority of both adult and pediatric patients held non-agricultural household registrations, accounting for 73.9 and 68.5%, respectively. The most common patient origins were from East China and South China, constituting more than half of the total patient population. The majority of patients had an annual household income below 200,000 RMB, with 70.37% of adult patients and 71.29% of pediatric patients falling into this category. Among the included adult patients, 52.2% had experienced their first onset of the disease more than 10 years ago, whereas the onset time for pediatric patients was predominantly between 1 and 5 years, accounting for 63.3%. The study found that the majority of pediatric AD cases were among children in elementary school and below. The pediatric patients were categorized based on their current study stage: 569 (94.99%) were in elementary school and below, 22 (3.67%) were in junior high school, while only 8 (1.34%) were in senior high school/technical school/vocational school.

**Table 2:** Demographic characteristics of adult patients and pediatric parents.

Variable	Adult patients (*n* = 594)	Pediatric patients (*n* = 599)
Age (years, Mean ± SD)	31.92 ± 11.79	5.50 ± 3.44
Gender (*n*, %)		
Male	246, 41.41%	314, 52.42%
Female	348, 58.59%	285, 47.58%
Highest education level/current study stage (*n*, %)		
Elementary School and Below	3, 0.51%	569, 94.99%
Junior High School	27, 4.55%	22, 3.67%
Senior High School/Technical School/Vocational School	84, 14.14%	8, 1.34%
Undergraduate/College	422, 71.04%	0
Master’s Degree and Above	58, 9.76%	0
Household registration type (*n*, %)		
Non-Agricultural Household	439, 73.91%	410, 68.45%
Agricultural Household	155, 26.09%	189, 31.55%
Region of residence (*n*, %)		
Northeast China	39, 6.51%	39, 6.57%
North China	40, 6.68%	75, 12.63%
Central China	109, 18.20%	81, 13.64%
East China	214, 35.73%	168, 28.28%
South China	127, 21.20%	165, 27.78%
Northwest China	17, 2.84%	21, 3.54%
Southwest China	53, 8.85%	45, 7.58%
Annual household income in the past year (*n*, %)		
Below 100,000 RMB	231, 38.89%	190, 31.72%
100,000–200,000 RMB	187, 31.48%	237, 39.57%
200,000–300,000 RMB	80, 13.47%	87, 14.52%
300,000–400,000 RMB	45, 7.58%	48, 8.01%
400,000–500,000 RMB	23, 3.87%	10, 1.67%
Above 500,000 RMB	28, 4.71%	27, 4.51%
Time since first onset (*n*, %)		
Less than 1 year	23, 3.87%	64, 10.68%
1–5 years	145, 24.41%	379, 63.27%
5–10 years	116, 19.53%	125, 20.87%
10 years or more	310, 52.19%	31, 5.18%

### 3.2 Scores comparison

Table 3 compares knowledge, attitude, and practice scores between adult patients and parents of pediatric patients. Adult patients generally have higher knowledge scores, particularly in bathing and skincare (3.64 ± 0.68 vs. 3.35 ± 0.93, *F* = 65.479, *p* < 0.001) and treatment precautions (3.90 ± 0.36 vs. 3.81 ± 0.51, *F* = 50.550, *p* < 0.001), leading to a higher total knowledge score (10.16 ± 1.22 vs. 9.80 ± 1.45, *F* = 16.735, *p* < 0.001). Attitude scores are similar between the groups (0.73 ± 0.94 vs. 0.69 ± 0.96, *F* = 0.759, *p* = 0.384).

**Table 3:** Comparison of knowledge, attitude, and practice scores between adult patients and parents of pediatric patients.

Item	Adult patients (*n* = 594)	Pediatric patients (*n* = 599)	*F*	*t*
	Mean ± SD			
Disease characteristics	2.62 ± 0.64	2.65 ± 0.63	1.050	0.306
Bathing and skincare	**3.64 ± 0.68**	**3.35 ± 0.93**	**65.479**	**0.000**
Treatment precautions	**3.90 ± 0.36**	**3.81 ± 0.51**	**50.550**	**0.000**
Total knowledge score	**10.16 ± 1.22**	**9.80 ± 1.45**	**16.735**	**0.000**
Total attitude score	0.73 ± 0.94	0.69 ± 0.96	0.759	0.384
Treatment adherence behavior	**3.50 ± 1.04**	**3.33 ± 1.19**	**15.687**	**0.000**
Self-treatment behavior	1.62 ± 1.29	1.49 ± 1.26	0.615	0.433
Lifestyle management behavior	**1.85 ± 0.39**	**1.69 ± 0.59**	**128.505**	**0.000**
Risk avoidance behavior	**1.58 ± 0.79**	**1.62 ± 0.88**	**8.059**	**0.005**
Total practice score	8.54 ± 2.20	8.13 ± 2.37	2.291	0.130

Bold value means statistical significance.

In terms of practices, adult patients score higher in treatment adherence (3.50 ± 1.04 vs. 3.33 ± 1.19, *F* = 15.687, *p* < 0.001) and lifestyle management (1.85 ± 0.39 vs. 1.69 ± 0.59, *F* = 128.505, *p* < 0.001), while parents score slightly higher in risk avoidance (1.62 ± 0.88 vs. 1.58 ± 0.79, *F* = 8.059, *p* = 0.005). There is no significant difference in self-treatment practices (1.62 ± 1.29 vs. 1.49 ± 1.26, *F* = 0.615, *p* = 0.433) or total practice scores (8.54 ± 2.20 vs. 8.13 ± 2.37, *F* = 2.291, *p* = 0.130). These findings suggest that adult patients are more knowledgeable and proactive in some health practices, while parents excel in risk avoidance.

### 3.3 Knowledge

Table 4 compares knowledge scores between adult patients and parents of pediatric patients. The average knowledge score for adult patients was 9.80 ± 1.45, while for parents of pediatric patients, it was 10.16 ± 1.22. The difference in knowledge scores was statistically significant (Z = −4.512, *p* < 0.001).

**Table 4:** Comparison of knowledge scores between adult patients and parents of pediatric patients.

	Adult patients (*n* = 594)				Pediatric patients (*n* = 599)			
	*n*	Mean ± SD	*Z*	*p*	*n*	Mean ± SD	*Z*	*p*
**Gender**			**3.109**	**0.002**			−0.980	0.327
Male	246	9.60 ± 1.51			314	10.17 ± 1.28		
Female	348	9.95 ± 1.40			285	10.14 ± 1.14		
**Highest education level/current study stage**			8.692	0.122			1.645	0.439
Elementary School and Below	3	9.33 ± 1.53			569	10.15 ± 1.22		
Junior High School	27	10.00 ± 1.11			22	10.41 ± 1.05		
Senior High School/Technical School/Vocational School	84	9.29 ± 1.91			8	10.00 ± 1.31		
Undergraduate/College	121	9.80 ± 1.42			0	-		
Master’s Degree and Above	301	9.89 ± 1.35			0	-		
Elementary School and Below	58	10.02 ± 1.28			0	-		
**Household registration type**			0.082	0.935			**−3.256**	**<0.001**
Non-Agricultural Household	439	9.82 ± 1.42			410	10.29 ± 1.01		
Agricultural Household	155	9.75 ± 1.55			189	9.87 ± 1.54		
**Region of residence**			4.495	0.610			2.056	0.915
Northeast China	39	9.67 ± 1.71			39	10.13 ± 1.06		
North China	75	9.96 ± 1.28			40	10.15 ± 1.81		
Central China	168	9.86 ± 1.45			214	10.19 ± 1.16		
East China	165	9.85 ± 1.44			127	10.20 ± 1.18		
South China	81	9.54 ± 1.64			109	10.11 ± 1.17		
Northwest China	21	9.62 ± 1.32			17	10.00 ± 1.22		
Southwest China	45	9.82 ± 1.27			53	10.08 ± 1.24		
**Annual household income in the past year**			10.701	0.058			4.288	0.509
Below 100,000 RMB	231	9.68 ± 1.53			190	10.16 ± 1.18		
100,000–200,000 RMB	187	9.76 ± 1.44			237	10.10 ± 1.15		
200,000–300,000 RMB	80	9.85 ± 1.54			87	10.16 ± 1.52		
300,000–400,000 RMB	45	10.13 ± 0.97			48	10.29 ± 1.11		
400,000–500,000 RMB	23	9.83 ± 1.34			10	10.10 ± 1.29		
Above 500,000 RMB	28	10.39 ± 1.20			27	10.37 ± 1.11		
**Time since first onset**			3.434	0.329			2.755	0.431
Less than 1 year	23	9.74 ± 1.60			64	10.08 ± 1.19		
1–5 years	145	9.57 ± 1.69			379	10.12 ± 1.26		
5–10 years	116	9.79 ± 1.37			125	10.26 ± 1.16		
10 years or more	310	9.92 ± 1.35			31	10.35 ± 1.02		

Bold value means statistical significance.

Among 594 adult patients, knowledge scores differed significantly by gender (*p* < 0.05), with females scoring higher (9.95 ± 1.40) than males (9.60 ± 1.51). No significant differences were found for other demographics. For 599 parents of pediatric patients, knowledge scores differed significantly by household registration type (*p* < 0.001), with non-agricultural households scoring higher (10.29 ± 1.01) than agricultural households (9.87 ± 1.54). No significant differences were found for other demographics (Table 4).

### 3.4 Attitudes

The average attitude score for adult patients was 0.69 ± 0.96, while the average attitude score for parents of pediatric patients was 0.73 ± 0.94. The difference in the distribution of attitude scores between these two groups was not statistically significant (Z = −1.459, *p* = 0.145).

Among the 594 adult patients, only the difference in attitude scores by household registration type was statistically significant (Z = −1.999, *p* = 0.046). For the 599 parents of pediatric patients, the differences in attitude scores by household registration type (*p* = 0.003) and annual household income (*p* < 0.001) were statistically significant (Table 5).

**Table 5:** Comparison of attitudes scores between adult patients and parents of pediatric patients.

	Adult patients (*n* = 594)				Pediatric patients (*n* = 599)			
	*n*	Mean ± SD	*Z*	*p*	*n*	Mean ± SD	*Z*	*p*
**Gender**			−0.24	0.981			−0.312	0.755
Male	246	0.7 ± 0.98			314	0.74 ± 0.93		
Female	348	0.68 ± 0.95			285	0.73 ± 0.96		
**Highest education level/current study stage**			7.059	0.216			0.063	0.969
Elementary School and Below	3	1.67 ± 1.53			569	0.74 ± 0.95		
Junior High School	27	0.41 ± 0.84			22	0.64 ± 0.79		
Senior High School/Technical School/Vocational School	84	0.63 ± 0.89			8	0.75 ± 1.04		
Undergraduate/College	121	0.76 ± 1.05			0	-		
Master’s Degree and Above	301	0.71 ± 0.97			0	-		
Elementary School and Below	58	0.55 ± 0.88			0	-		
**Household Registration Type**			**−1.999**	**0.046**			**−2.98**	**0.003**
Non-Agricultural Household	439	0.73 ± 0.99			410	0.81 ± 0.97		
Agricultural Household	155	0.56 ± 0.89			189	0.57 ± 0.86		
**Region of residence**			2.012	0.919			11.147	0.084
Northeast China	39	0.59 ± 0.94			39	0.79 ± 1		
North China	75	0.64 ± 0.83			40	0.6 ± 0.74		
Central China	168	0.71 ± 1.03			214	0.86 ± 0.97		
East China	165	0.73 ± 0.99			127	0.62 ± 0.93		
South China	81	0.68 ± 0.93			109	0.65 ± 0.94		
Northwest China	21	0.43 ± 0.75			17	0.47 ± 0.72		
Southwest China	45	0.71 ± 1.01			53	0.77 ± 1.01		
**Annual household income in the past year**			7.948	0.159			**43.015**	**<0.001**
Below 100,000 RMB	231	0.59 ± 0.9			190	0.5 ± 0.85		
100,000–200,000 RMB	187	0.67 ± 0.95			237	0.7 ± 0.91		
200,000–300,000 RMB	80	0.81 ± 1.07			87	1.24 ± 1.06		
300,000–400,000 RMB	45	0.71 ± 0.97			48	0.83 ± 0.88		
400,000–500,000 RMB	23	0.96 ± 1.11			10	0.7 ± 0.95		
Above 500,000 RMB	28	0.96 ± 1.04			27	0.81 ± 1.04		
**Time since first onset**			4.909	0.179			1.512	0.680
Less than 1 year	23	0.7 ± 0.88			64	0.66 ± 0.98		
1–5 years	145	0.59 ± 0.89			379	0.74 ± 0.95		
5–10 years	116	0.66 ± 1.06			125	0.76 ± 0.94		
10 years or more	310	0.74 ± 0.97			31	0.74 ± 0.86		

Bold value means statistical significance.

### 3.5 Practices

The average practice score for adult patients was 8.14 ± 2.37, while the average practice score for parents of pediatric patients was 8.54 ± 2.20. The difference in the distribution of practice scores between these two groups was statistically significant (Z = -2.720, *p* = 0.007).

Among the 594 adult patients, statistically significant differences in practice scores were found based on the highest education level (Z = 35.942, *p* < 0.001), household registration type (Z = 2.448, *p* = 0.014). Adult patients with the highest education level of junior high school had the highest practice scores, followed by those with senior high school/technical school and master’s degree. Details can be found in Table 6.

**Table 6:** Comparison of practices scores between adult patients and parents of pediatric patients.

	Adult patients (*n* = 594)			Pediatric patients (*n* = 599)				
	*n*	Mean ± SD	*Z*	*p*	*n*	Mean ± SD	*Z*	*p*
Gender			−1.722	0.085			−1.774	0.076
Male	246	8.28 ± 2.49			314	8.69 ± 2.18		
Female	348	8.03 ± 2.29			285	8.38 ± 2.21		
**Highest education level/current study stage**			**35.942**	**<0.001**			5.132	0.077
Elementary School and Below	3	6.33 ± 1.53			569	8.50 ± 2.20		
Junior High School	27	9.52 ± 2.14			22	9.32 ± 1.86		
Senior High School/Technical School/Vocational School	84	9.21 ± 1.95			8	9.63 ± 2.07		
Undergraduate/College	121	8.01 ± 2.34			0	-		
Master’s Degree and Above	301	7.79 ± 2.39			0	-		
Elementary School and Below	58	8.05 ± 2.46			0	-		
**Household registration type**			**2.448**	**0.014**			−0.882	0.0378
Non-Agricultural Household	439	8.00 ± 2.36			410	8.57 ± 2.23		
Agricultural Household	155	8.52 ± 2.37			189	8.48 ± 2.12		
**Region of residence**			4.682	0.585			10.838	0.094
Northeast China	39	7.49 ± 2.71			39	8.18 ± 2.20		
North China	75	8.24 ± 2.35			40	8.08 ± 2.10		
Central China	168	8.39 ± 2.22			214	8.49 ± 2.22		
East China	165	8.22 ± 2.28			127	9.00 ± 2.09		
South China	81	7.90 ± 2.55			109	8.29 ± 2.29		
Northwest China	21	8.05 ± 2.42			17	8.53 ± 2.32		
Southwest China	45	7.71 ± 2.62			53	8.81 ± 2.07		
**Annual household income in the past year**			8.853	0.115			4.727	0.450
Below 100,000 RMB	231	8.47 ± 2.25			190	8.57 ± 2.18		
100,000–200,000 RMB	187	7.86 ± 2.37			237	8.35 ± 2.28		
200,000–300,000 RMB	80	8.03 ± 2.32			87	8.95 ± 1.80		
300,000–400,000 RMB	45	7.82 ± 2.64			48	8.48 ± 2.34		
400,000–500,000 RMB	23	8.00 ± 2.66			10	8.20 ± 2.62		
Above 500,000 RMB	28	8.14 ± 2.70			27	8.93 ± 2.32		
**Time since first onset**			5.753	0.124			1.935	0.586
Less than 1 year	23	9.00 ± 1.24			64	8.70 ± 2.35		
1–5 years	145	8.32 ± 2.43			379	8.45 ± 2.14		
5–10 years	116	8.07 ± 2.39			125	8.67 ± 2.29		
10 years or more	310	8.01 ± 2.40			31	8.77 ± 2.25		

Bold value means statistical significance.

### 3.6 Coefficients of factors

Regression analysis in Table 7 reveals significant factors influencing health practices among adult patients and parents of pediatric patients. Treatment adherence is positively affected by knowledge of treatment precautions (*t* = 4.316, *p* < 0.001) and skincare (*t* = 2.006, *p* = 0.045), but negatively by knowledge of disease characteristics (*t* = −2.109, *p* = 0.035). Self-treatment practices are positively associated with knowledge of disease characteristics (*t* = 2.559, *p* = 0.011) and attitudes towards medication (*t* = 7.385, *p* < 0.001), with no influence from patient type.

**Table 7:** Coefficients of factors influencing practices (*n* = 1,193).

	Unstandardized coefficients		Standardized		
	B	Std. error	Beta	*t*	Sig.
Treatment adherence					
Knowledge of disease characteristics	**−0.112**	**0.053**	**−0.064**	**−2.109**	**0.035**
Knowledge of bathing and skincare	**0.082**	**0.041**	**0.061**	**2.006**	**0.045**
Knowledge of treatment precautions	**0.323**	**0.075**	**0.129**	**4.316**	**0.000**
Attitude towards medication	−0.026	0.034	−0.022	−0.766	0.444
Patient type	−0.104	0.065	−0.047	−1.589	0.112
Household registration type	0.073	0.071	0.030	1.020	0.308
Self-treatment					
Knowledge of disease characteristics	**0.154**	**0.060**	**0.076**	**2.559**	**0.011**
Knowledge of bathing and skincare	−0.045	0.046	−0.029	−0.971	0.332
Knowledge of treatment precautions	0.082	0.085	0.029	0.965	0.335
Attitude towards medication	**0.282**	**0.038**	**0.210**	**7.385**	**0.000**
Patient type	−0.116	0.074	−0.045	−1.565	0.118
Household registration type	0.120	0.081	0.043	1.495	0.135
Lifestyle management					
Knowledge of disease characteristics	0.012	0.024	0.015	0.508	0.611
Knowledge of bathing and skincare	**0.071**	**0.018**	**0.117**	**3.921**	**0.000**
Knowledge of treatment precautions	**0.160**	**0.033**	**0.141**	**4.810**	**0.000**
Attitude towards medication	−0.003	0.015	−0.005	−0.184	0.854
Patient type	**−0.127**	**0.029**	**−0.126**	**−4.381**	**0.000**
Household registration type	0.011	0.032	0.010	0.338	0.735
Risk avoidance					
Knowledge of disease characteristics	−0.011	0.040	−0.008	−0.271	0.787
Knowledge of bathing and skincare	**0.128**	**0.031**	**0.126**	**4.149**	**0.000**
Knowledge of treatment precautions	**0.163**	**0.056**	**0.087**	**2.905**	**0.004**
Attitude towards medication	−0.032	0.025	−0.036	−1.251	0.211
Patient type	**0.101**	**0.049**	**0.060**	**2.053**	**0.040**
Household registration type	0.087	0.054	0.047	1.619	0.106
Total practice score					
Total knowledge score	**0.227**	**0.069**	**0.094**	**3.300**	**0.001**
Total attitude score	**0.265**	**0.049**	**0.156**	**5.415**	**0.000**
Patient type	**0.310**	**0.145**	**0.061**	**2.134**	**0.033**
Household registration type	**−0.287**	**0.132**	**−0.063**	**−2.179**	**0.030**

Bold value means statistical significance.

Lifestyle management benefits from knowledge of skincare (*t* = 3.921, *p* < 0.001) and treatment precautions (*t* = 4.810, *p* < 0.001) but is negatively associated with patient type (*t* = −4.381, *p* < 0.001). Risk avoidance is positively influenced by knowledge of skincare (*t* = 4.149, *p* < 0.001), treatment precautions (*t* = 2.905, *p* = 0.004), and patient type (*t* = 2.053, *p* = 0.040).

Total practice score is positively influenced by overall knowledge (*t* = 3.300, *p* = 0.001) and attitude (*t* = 5.415, p < 0.001), with patient type having a positive (*t* = 2.134, *p* = 0.033) and household registration type a negative association (*t* = −2.179, *p* = 0.030).

## 4 Discussion

The aim of the study is to identify the factors that influence AD patients’ practices regarding disease management in China. This study reveals that overall knowledge and attitude significantly is positively associated with practice, consistent with the study’s hypothesis.

These results are consistent with the study’s hypothesis. The findings indicate that enhancing patients’ knowledge and attitudes toward AD management is crucial, which suggests that future research should focus on identifying appropriate interventions to improve patients’ cognition and attitudes toward AD management, a point that has been confirmed by previous studies (11).

This study found that adult patients scored higher in knowledge than parents of pediatric patients. This may be because individuals with a history of the disease have direct experience in managing it, leading to a better understanding of AD (20). The attitude scores for adult patients and parents of pediatric patients were similar, indicating that both groups hold similar attitudes toward AD.

In practice, adult patients scored significantly higher in treatment adherence and lifestyle management practices, reflecting that adult patients are more actively involved in managing their health compared to parents managing their children’s health. Also, studies found that among pediatric patients, medication cost and concerns about side effects are the most common reasons for nonadherence (24, 25). Other factors such as parents’ cooperation, resentment against treatment, reluctance to bathe, and late bedtimes may contribute to management issues in practice for pediatric patients, resulting in lower scores (26, 27). However, parents of pediatric patients scored slightly higher in risk avoidance practices, likely due to their heightened attention and protective measures regarding their children’s health, resulting in greater attention to details in daily life.

The regression analysis results indicate that treatment adherence behavior is positively associated with knowledge of treatment precautions and bathing and skincare knowledge, while knowledge of disease characteristics has a negative impact. This suggests that patients with more knowledge about the disease may have poorer adherence, which is inconsistent with the view of Patel et al. (28). This discrepancy may be because patients who know more about the disease are more likely to adopt new treatment methods, leading to poorer adherence to traditional medical advice. Self-treatment behavior is positively influenced by knowledge of disease characteristics and medication attitudes. This may be because patients with more knowledge about the disease and a more open attitude towards medication are more likely to use new medications spontaneously, believing they understand the disease well enough to adjust dosages on their own.

Lifestyle management is negatively associated with patient type, indicating that adult patients have better lifestyle management behaviors, while their risk avoidance behaviors are poorer. This may be related to adult patients having a better understanding of their condition. The strong correlation between knowledge of bathing, skincare, and treatment precautions with both practices suggests that patients who focus on treatment are more likely to adopt protective measures in daily life.

Based on the study’s findings, which indicate the positive effect of knowledge and attitude levels on patients’ management practice, efforts have been made to enhance patient knowledge and attitudes. At the policy level, the management of AD patients in China mainly focuses on medical insurance, disease prevention, and health education. In recent years, the government has issued a series of policy documents on the management of chronic diseases, aiming to improve the prevention, diagnosis, and treatment of chronic diseases (29). In China, several studies have demonstrated the benefits of patient education for managing AD, utilizing methods such as verbal instruction by outpatient physicians, new media, and the Inverted Classroom model (30–33). However, these studies are still at the pilot stage and have not been implemented nationwide, resulting in inconsistent patient education quality across hospitals. It was not until 2022 that the Chinese Society of Dermatology Immunology Group introduced the “Consensus on the Full Management of Atopic Dermatitis,” which was officially published in 2023 (34). Before this, China only had basic diagnostic and treatment guidelines for AD (35), and there was insufficient recognition of the specific management needs of AD patients at the individual level. The policy guidance on the management of AD is still in its early stages and needs further strengthening in the future.

There are some limitations to this study. The research sample was derived from an online questionnaire survey conducted by the online organization, which may introduce selection bias. Patients who participated in the survey might be more actively engaged in disease management, while those who did not participate could have different KAP statuses. Therefore, the results may not fully represent the situation of all AD patients. Additionally, since the data were collected using online questionnaires, clinical manifestations and severity of dermatitis were not accounted for in the study. Although the questionnaire was developed based on multiple articles and expert opinions, its internal consistency was only within an acceptable range (none of the Cronbach’s alpha of each dimension is above 0.7). This indicates that there is still room for improvement in the questionnaire’s evaluation of KAP. As a cross-sectional study, this research can only describe the KAP status at a specific point in time and cannot infer causality. Future research could adopt a longitudinal design to track changes in patients’ KAP and their impact on disease management outcomes, thereby better understanding the long-term effects of knowledge and attitudes on practices.

## Data Availability

The raw data supporting the conclusions of this article will be made available by the authors, without undue reservation.
